# Developing Innovative Cement Composites Containing Vine Shoot Waste and Superplasticizers

**DOI:** 10.3390/ma16155313

**Published:** 2023-07-28

**Authors:** Daniela Alexandra Scurtu, Leontin David, Erika Andrea Levei, Dorina Simedru, Xenia Filip, Cecilia Roman, Oana Cadar

**Affiliations:** 1 Research Institute for Analytical Instrumentation Subsidiary, National Institute for Research and Development for Optoelectronics INOE 2000, 67 Donath Street, 400293 Cluj-Napoca, Romania; erika.levei@icia.ro (E.A.L.); dorina.simedru@icia.ro (D.S.); cecilia.roman@icia.ro (C.R.); oana.cadar@icia.ro (O.C.); 2 Faculty of Physics, Babes-Bolyai University, 1 Mihail Kogalniceanu Street, 400084 Cluj-Napoca, Romania; 3 National Institute for Research and Development of Isotopic and Molecular Technologies, 67-103 Donath Street, 400293 Cluj-Napoca, Romania; xenia.filip@itim-cj.ro

**Keywords:** cement paste, vine shoot waste, superplasticizers, structural properties, NMR spectroscopy, mechanical strength

## Abstract

The expansion of the construction industry requires large quantities of construction materials; therefore, the utilization of alternative raw materials that reduce the environmental impact and enhance the quality of the construction materials has received increasing interest. The comparative performance of 1% Dynamon SR3 or Dynamon SR41 superplasticizers on the properties of cement paste with 1 wt.% vine shoot waste addition (VSW) was investigated after 28 days using Fourier-transform infrared (FT-IR) spectroscopy, X-ray diffraction (XRD), scanning electron microscopy with energy dispersive X-ray spectroscopy (SEM-EDX), and solid-state ^27^Al and ^29^Si nuclear magnetic resonance (NMR) spectroscopy. VSW does not delay the formation of calcium hydroxide and C–S–H and a slight decrease of the –OH band in samples containing superplasticizers, suggesting that free lime is converted to carbonates. The highest degree of crystallinity was remarked for the samples with superplasticizers. The structure of the cement paste with VSW and superplasticizers was corroborated with mechanical properties, showing increased strength in using VSW and superplasticizers. The results showed that adding 1% VSW and superplasticizers does not change the performance of the cement paste but reduces the water-cement ratio. The combination of VSW and superplasticizers led to cement composite with improved structural and mechanical properties suitable for construction.

## 1. Introduction

The expansion of the global economy and increased living standards led to the construction industry boom, which has further led to high demand for resources, materials shortages, supply chain disruptions, and an increase in construction material prices. The raw material demand and use are expected to increase annually and double by 2060 [1]. Production of construction materials is mainly based on non-regenerable raw materials, of which non-metallic minerals such as sand, gravel, limestone, and crushed rock account for more than half of the total materials consumed. The intensive use of non-regenerable raw materials has put high pressure on the environment. To reduce production costs and negative environmental impact, alternative raw materials and the incorporation of locally available solid wastes, such as demolition waste, plastic, glass, or lignocellulosic waste in cementitious materials, were recently considered [2].

The use of recycled concrete powder and recycled aggregate resulting from construction and demolition waste shows multiple advantages: decrease of the carbon footprint of the construction industry, reduction of the land surface used for landfills, and preservation of aggregate natural resources [3,4,5,6]. Plastic waste is another recycled aggregate that can be used successfully in self-compacting concrete. Recycled plastic waste can enhance sound insulation, fiber reinforcement, and thermal insulation [7,8,9]. Glass waste can also be used in cement-based materials, such as fine and coarse aggregate, as they increase cement-based materials’ flowability and compressive strength. By increasing the content of the glass waste, the bulk density, the water absorption, and the slump value decrease [10,11]. A better connection between the glass waste and the cement matrix is obtained by heating the glass waste to 600–800 °C [12,13].

Lignocellulosic biomass has a complex structure formed by polysaccharides (cellulose, hemicellulose, and holocellulose), lignin, and polar and non-polar substances [14,15]. Lignocellulosic waste from forestry and agriculture contains coir, cork granules, rice straw, hazelnut shell, rice straw, bark, arhar stalks, oil palm residue, and bagasse, and it is a promising raw material for the manufacturing of new cement-based materials due to the low costs, abundance, energy efficiency, and positive environmental impact [14,16]. The addition of rice husk and rice husk ash to cementitious materials enhances the mechanical properties by working as fillers in the cement matrix [17]. The fresh cementitious composite’s workability increases when corn straw fiber is treated with sodium hydroxide [18]. Positive effects on density, porosity, water absorption, toughness, and strength are obtained due to the better fiber-matrix interfacial adhesion between the cement matrix and treated short fibers of sugarcane bagasse [19].

Vine is an important crop all over the world [14]. Cane and vine shoot pruning, leaf trimming, and cluster thinning during cultivation and harvesting of grapes generate annually about 5 tons/hectare of solid waste consisting of grape stalks, unripe grapes, vine shoots, and vine canes and leaves [20]. Vine shoot waste (VSW) contains important amounts of lignocellulosic waste, but presently is underexploited, most frequently being field-burned, or used for compost production [21,22], yet it could be valorized for particle boards, cellulose nanocrystals, bioenergy, clay bricks, and cement-based materials’ production [23,24,25,26]. Presently, none of the VSW valorization routes are applicable on an industrial scale.

The use of waste in concrete formulations can increase the water requirements. High water content can have a negative effect on the structural characteristics of the cement-based materials. In this regard, superplasticizers enhance the properties of cement-based materials by reducing the water to cement ratio and increasing the workability, producing higher workability at the same or lower water content [27]. The superplasticizers use electrostatic and/or steric forces to oppose attractive forces between cement grains and reduce their yield stress [28]. The main factors that influence the effect of superplasticizers on cement-based materials are their molecular structure, environmental conditions, type, cement composition, dosage, and compatibility between cement and superplasticizers [29]. Superplasticizers represent a key ingredient in producing high-strength cement-based materials [30]. By using superplasticizers, the cement content and water requirement in cement-based materials are reduced, the durability is enhanced [31]. Among superplasticizers, naphthalene sulfonate, polycarboxylate, and melamine-sulfonated superplasticizers used in biomass-activated grouts decrease the cement-based materials’ initial setting time [32]. Acrylic polymers (with no formaldehyde) in a water solution (Dynamon SR3–SR3 and Dynamon SR41–SR41) used in cement-based materials increase their initial setting time [33]. The cement hydration process is divided into five stages: initial, dormant, hardening, cooling, and densification. The densification is from 20 h and lasts as long as the cement grains are in contact with water. After about 28 days, the hydration of the cement is considered closed as most of the cement grains have reacted with water, forming hydration products [33,34,35,36].

This study aims to investigate the enhancement of the structure of cement paste (CP) containing 1% VSW by adding 1% superplasticizers to lower the water-cement ratio. The structure of the CP with VSW and superplasticizers was evidenced using Fourier-transform infrared (FT-IR) spectroscopy, X-ray diffraction (XRD), scanning electron microscopy with energy dispersive X-ray analysis (SEM-EDX) and solid-state ^27^Al and ^29^Si nuclear magnetic resonance (NMR) spectroscopy. The mechanical performance (flexural strength and compressive strength) of the CP samples was also investigated.

## 2. Materials and Methods

### 2.1. Sample Preparation

White Portland cement was used in this study as it contains a lower quantity of iron (0.5% Fe_2_O_3_) than the grey cement (5% Fe_2_O_3_). A high Fe content has a negative effect on the NMR measurements due to the use of magnetic fields and radio impulses. Portland cement is one of the purest types of cement and has a high content of clinker (95–100 wt.%), and low content of auxiliary components (0–5 wt.%). The cement used in this study has the following composition: 65.20 wt.% CaO, 21.73 wt.% SiO_2_, 4.63 wt.%, Al_2_O_3_, 0.45 wt.% Fe_2_O_3_, 1.00 wt.% MgO, 0.36 wt.% Na_2_O, 0.14 wt.% K_2_O, 3.00 wt.% SO_3_, and loss of ignition is 0.1 wt.%, which is in accordance with EN 197-1 [37] and EN 196-2 standards [38].

Commercially available superplasticizers containing water-based solution of acrylic polymers with no formaldehyde SR3 and SR41, produced by Mapei, Italy, were used in the experiments. Superplasticizers were added to disperse the cement grain and favor the low hydration process. VSW was procured from a private garden. First, the VSW was dried at 105 °C for 24 h and, after cooling, was ground to powder (<100 µm). All chemicals (Merck, Darmstadt, Germany) have analytical grade and were used without purification. Samples were prepared by adding the distilled water and superplasticizer mixture to the cement grains or to the homogeneous dry mixture of cement grains and VSW, as presented in Table 1. The water-to-cement ratio for all samples was 0.3, except CPW. The sample ingredients were mixed using an electric mixer with a rotational frequency of 700 rpm for 5 min, and after mixing, they were poured into a standard mold. The standard mold was kept in a closed chamber at 20 °C and relative humidity (45%) conditions; after demolding, the specimens were removed from the molds and cured in water until the testing day.

### 2.2. Material Characterization

The FT-IR spectra were recorded using a Spectrum BX II (Perkin Elmer, Waltham, MA, USA) spectrometer in the range of 4000–400 cm^−1^ on 1% KBr pellets with a 2 cm^−1^ spectral resolution.

The XRD pattern was recorded on a D8 Advance diffractometer (Bruker, Karlsruhe, Germany) with CuKα1 radiation (λ = 1.5406 Å) operated at 40 kV and 35 mA on powder samples. The semi-quantitative evaluation was performed following the Reference Intensity Ratio (RIR) method [39]. The degree of crystallinity was calculated as the ratio between the area of diffraction peaks and the total area of diffraction peaks and amorphous halos.

A scanning electron microscope (VEGAS 3 SBU, Tescan, Brno-Kohoutovice, Czech Republic) with a Quantax EDX XFlash (Bruker, Karlsruhe, Germany) detector was used for the SEM-EDX analysis. The analysis was performed on small specimens of ~5 mm^2^.

^29^Si and ^27^Al MAS NMR spectra were recorded on a 500 MHz Bruker Advance III (Bruker BioSpin GmbH, Germany) solid-state wide-bore spectrometer, operating at ^29^Si and ^27^Al Larmor frequencies of 99.36 and 130.32 MHz, respectively. The ^29^Si MAS NMR spectra were recorded using a 4 mm Bruker MAS probe head, with samples being packed into ZrO_2_ rotors, which were spun at a rate of 7 kHz. It used one-pulse with a proton high-power decoupling sequence with a recycle delay of 5 s, collecting 15,000 FIDs for all powder samples. The ^29^Si spectra were calibrated to tetramethylsilane (TMS) through an indirect procedure that uses sodium-3-(trimethylsilyl)-propane-1-sulfonate (DSS) (1.46 ppm) as external reference. The ^27^Al MAS NMR spectra were recorded using a 2.5 mm Bruker MAS probe head, and the samples being packed into ZrO_2_ rotors were spun at a rate of 25 kHz. It used one-pulse sequence with a pulse width of 1.5 ms, recycle delay of 1 s, and accumulated 3000 FIDs. The ^27^Al spectra were calibrated to the signal (at 0 ppm) of aluminum in an external standard 1M aqueous solution of Al(NO_3_)_3_.

The mechanical properties of cement pastes were carried out on 40 mm × 40 mm × 160 mm prisms for the flexural strength and 40 mm × 40 mm × 40 mm cubed for the compressive strength (BS EN 196-1:2016, [40]) using a UTCM-3742 15/250 kN Automatic Cement Flexure/Compression Testing Machine (Utest Material Testing Equipment, Ankara, Turkey). To determine the flexural strength, the prism stays on two supported beams and from the opposite direction a beam loading forces the prism in the middle of the distance between the two supported beams; this load is also known as one-point loading. To determine the compressive strength, the sample stays on a fixed platen with dimensions of 40 mm × 40 mm × 40 mm and, from the opposite direction, a platen with the same dimension load force on the sample.

## 3. Results

The investigations were carried out at 28 days when the hydration of the cement is considered to be closed, most cement grains reacted with water and have formed hydration products around them. The use of VSW positively impacts the microstructure of the cement paste, but the water requirement is higher than in cement paste [27].

### 3.1. Fourier-Transform Infrared Spectroscopy (FT-IR)

**Figure 1:**
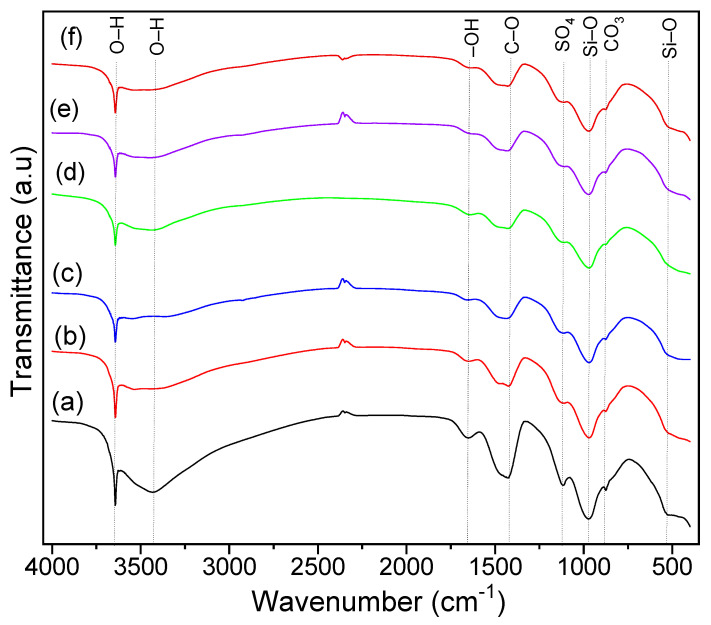
FT-IR spectra of (a) CP, (b) CPSR3, (c) CPSR41, (d) CPW, (e) CPSR3W, and (f) CPSR41W samples.

In the FT-IR spectra (Figure 1) of all samples, the presence of the intense bands specific to carbonate (∼880 and ∼1420 cm^−1^) and Si–O (∼530 and ∼960 cm^−1^) vibration indicate the presence of calcium silicate hydrate (C–S–H) gel, responsible for the strength of the concrete [41]. The intensity of C–O vibration in carbonates at ∼1420 cm^−1^ is proportional to the carbonation degree [42]. Carbonates are formed following the reaction of free lime (Ca(OH)_2_) with the CO_2_ from the atmosphere. The intensity of the peak attributed to absorbed water molecules (∼1650 cm^−1^ and ∼3420 cm^−1^) decreased in samples containing superplasticizers and VSW, compared to the CP. The peak at ∼1100 cm^−1^ corresponds to the vibration of sulfates (SO_4_^2−^) in ettringite [43]. The ∼3640 cm^−1^ band is specific to –OH stretching vibration in Ca(OH)_2_ [43]. The similar intensity of the peaks ∼960 cm^−1^ in the samples containing VSW and in CP confirms that the addition of VSW does not impede the formation of calcium hydroxide and C–S–H. Also, the slight decrease of the –OH band in samples containing plasticizer and plasticizer-waste mixture suggests that free lime is converted to carbonates. In all spectra, the doublet around 2350 cm^−1^ is due to traces of gaseous CO_2_ present in the sample compartment of the spectrometer.

### 3.2. X-ray Diffraction (XRD) Analysis

**Figure 2:**
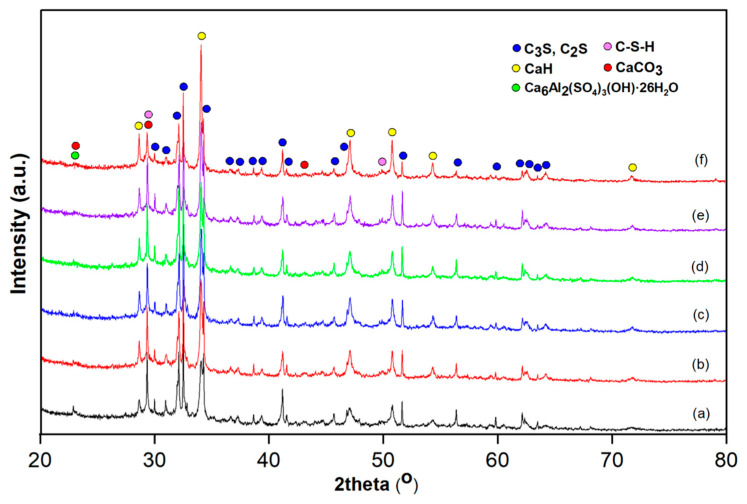
XRD patterns of (a) CP, (b) CPSR3, (c) CPSR41, (d) CPW, (e) CPSR3W, and (f) CPSR41W samples.

The XRD patterns of the CP, CPSR3, CPSR41, CPW, CPSR3W, and CPSR41W samples after 28 days of hydration displayed the anticipated hydration products, namely portlandite (calcium hydroxide, CH), calcite (CaCO_3_), poorly crystallized calcium silicate hydrate (C–S–H), and unreacted clinker phases (mainly calcium silicates) (Figure 2).

The composition of crystalline phases identified by RIR method of the investigated samples is presented in Table 2. The presence of calcite was attributed to the partial carbonation of portlandite by the reaction of CH with atmospheric CO_2_. Also, traces of ettringite were present in all studied samples, suggesting the occurrence of phase reversal. Regarding the anhydrous clinker phases, only C_2_S (belite) and C_3_S (alite) did not entirely react. After 28 days of hydration, the same unreacted and hydration products were observed in all the samples. A slightly lower intensity of C_2_S and C_3_S diffraction peaks can be observed in the charging effects and heterogeneous nucleation by adding VSW due to the higher consumption of C_2_S and C_3_S [27,44]. Generally, superplasticizers influence hydration by reducing the formation of CH and ettringite in the initial stages of curing, which can be remarked on by XRD. However, the XRD patterns of CPSR3, CPSR41, CPSR3W, and CPSR41W samples did not show any difference in hydration due to the incorporation of superplasticizers (SR3 and SR41), possibly due to the low content of superplasticizers used. Similar results were obtained by Chakkamalayath et al., who reported the performance of polycarboxylate (PCE) and naphthalene (SNF)-based superplasticizers on some properties of cement paste containing Type I and Type V cement and volcanic ash [45]. The degree of crystallinity (DC) of the investigated samples varies between 71.0 and 78.1%, the highest values being remarked for the samples with superplasticizers, e.g., CPSR3 (78.1%) and CPSR41 (76.2%) (Table 2).

### 3.3. Scanning Electron Microscopy with Energy Dispersive X-ray Spectroscopy (SEM-EDX)

**Figure 3:**
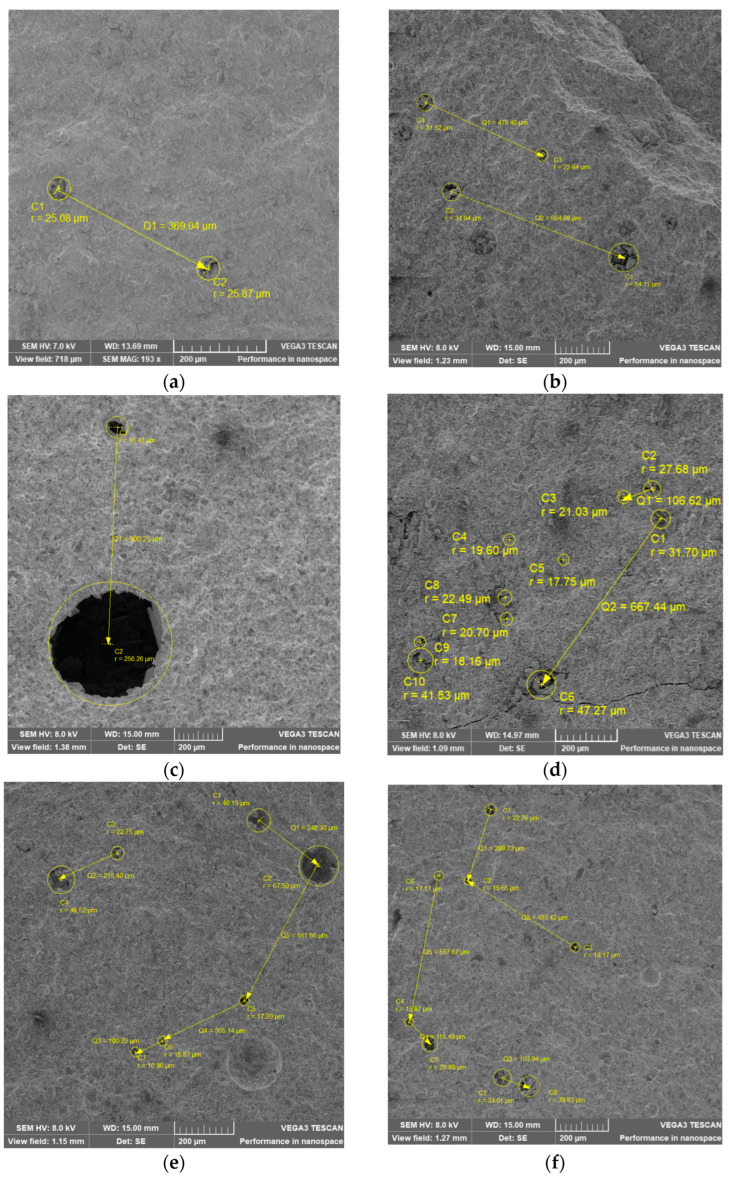
Air holes size for (**a**) CP, (**b**) CPSR3, (**c**) CPSR41, (**d**) CPW, (**e**) CPSR3W, and (**f**) CPSR41W samples.

SEM-EDX, a powerful surface investigation tool, was used to study the changes on the surface of cement paste due to the addition of superplasticizers and/or VSW. The homogeneity, defects, and variations in the chemical composition of the examined samples were followed. CP, CPSR3, CPSR41, CPW, CPSR3W, and CPSR41W samples were first investigated at medium magnification (100–250×). The obtained images are presented in Figure 3. All the samples under investigation appear to have a surface that is mostly homogeneous, with air holes of various sizes. Additionally, the incorporation of wood or the superplasticizer SR3 to CP (Figure 3b,d) appear to have an extra effect on the surface, resulting in fractures and forms that are dispersed randomly.

The pore structure of solidified cement is made up of air holes, gel pores, and small and large capillary pores [46]. The hydrated cement paste’s air holes occupy the empty spaces left by the solid components in the process of hydration [47]. Entrapped air voids (large as 3 mm) and entrained air voids (50 to 200 μm) play a significant role in the uses of cement since they have the potential to negatively impact its strength [47]. In addition to their unfavorable effects, entrained air voids are known to increase the freeze and thaw resistance of cementitious materials [48,49,50]. Due to the size of airholes (>several μm), SEM is one of the most useful techniques to observe and quantify them [46,51].

In case of CP, CPSR3, CPSR41, CPW, CPSR3W, and CPSR41W samples, the air holes from the sample surface and the distance between them were measured using Bruker Esprit 2.0 software (Bruker Nano GmbH, Berlin, Germany), as shown in Figure 3 and the results are presented in Table 3. The most uniform surface is the CP surface, which has a few air holes dispersed randomly throughout. The size and spacing between the air holes both expand with the addition of superplasticizer. The surface response to the addition of VSW to CP is similar to that of the addition of superplasticizer. Air holes expand and their spacing decreases as a result of the simultaneous addition of superplasticizer and VSW. Similar behavior has been observed in other admixed cement paste [52,53].

**Figure 4:**
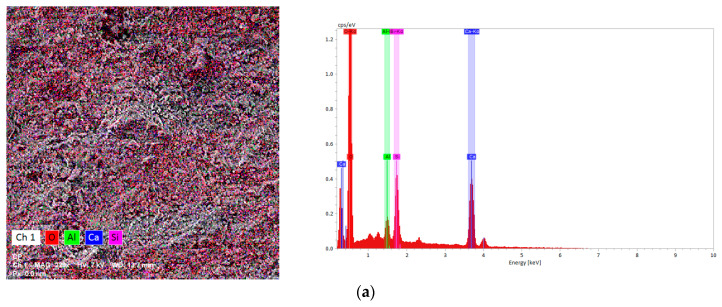

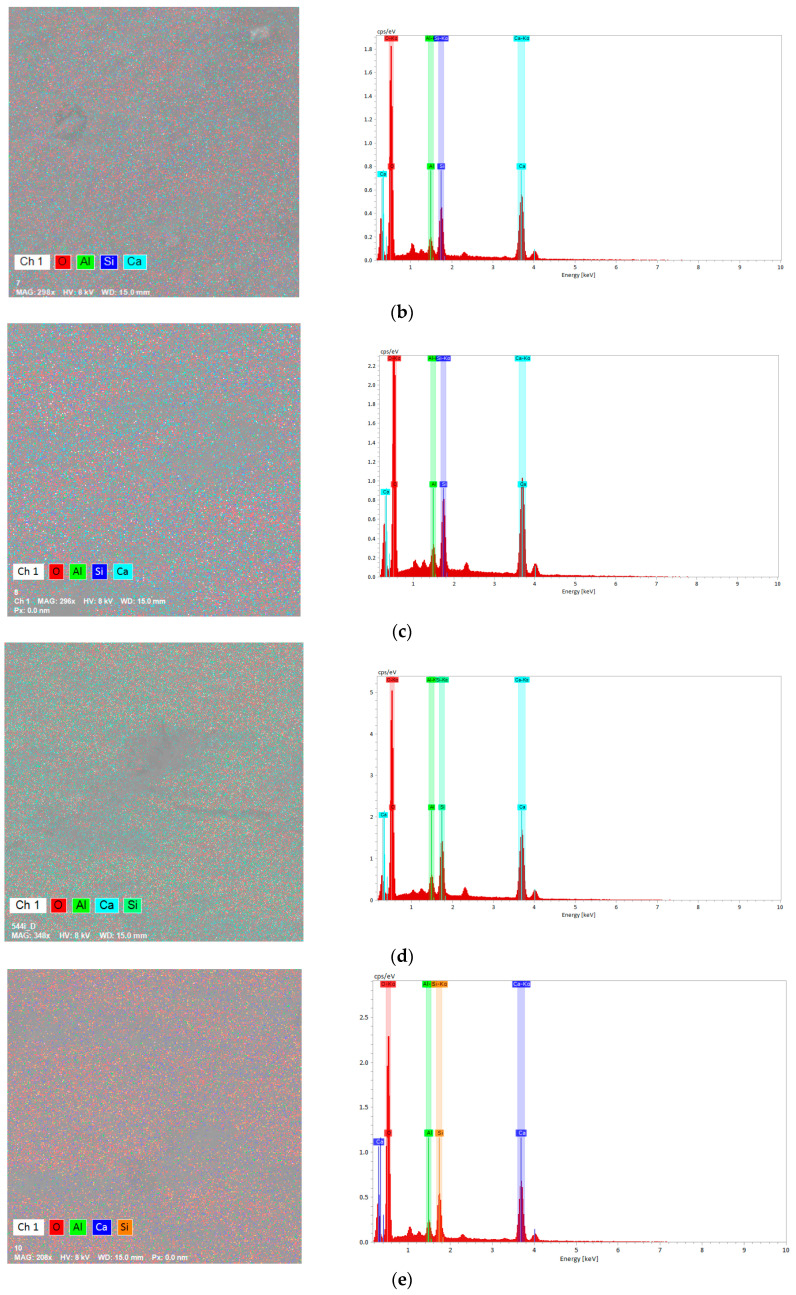

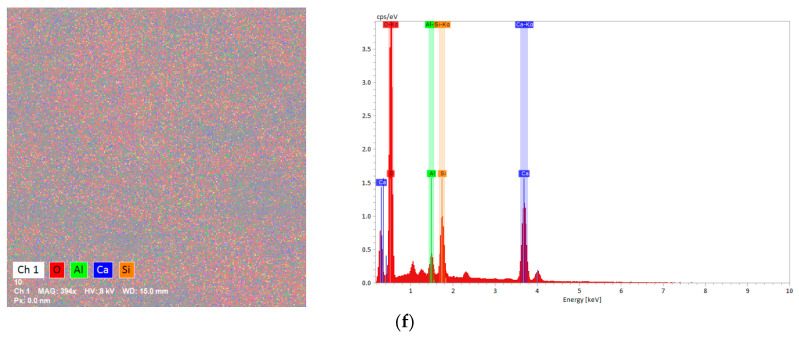
Surface mapping and EDX spectra of (**a**) CP, (**b**) CPSR3, (**c**) CPSR41, (**d**) CPW, (**e**) CPSR3W, and (**f**) CPSR41W samples.

The elemental analysis of the investigated samples was performed at higher magnification (208–394×) using the EDX detector. The images obtained, represented in “false colors”, are presented in Figure 4 and show a relative homogenous distribution of the compounds in all samples. The elemental composition is presented in Table 4. The C/S ratio was calculated for each sample. The values vary between 4.31 and 4.95. The main binding phase of Portland cement and concrete, known as C–S–H, is well-recognized for giving concrete its strength. In pure Portland cement, the C/S ratio for C–S–H ranges from 1.3 to 2.1 with a mean of 1.75 [54]. The values obtained for the studied samples’ C/S ratio that are greater than 2.1 support the findings from XRD patterns that showed the presence of other Ca-based compounds like calcite and portlandite.

**Figure 5:**
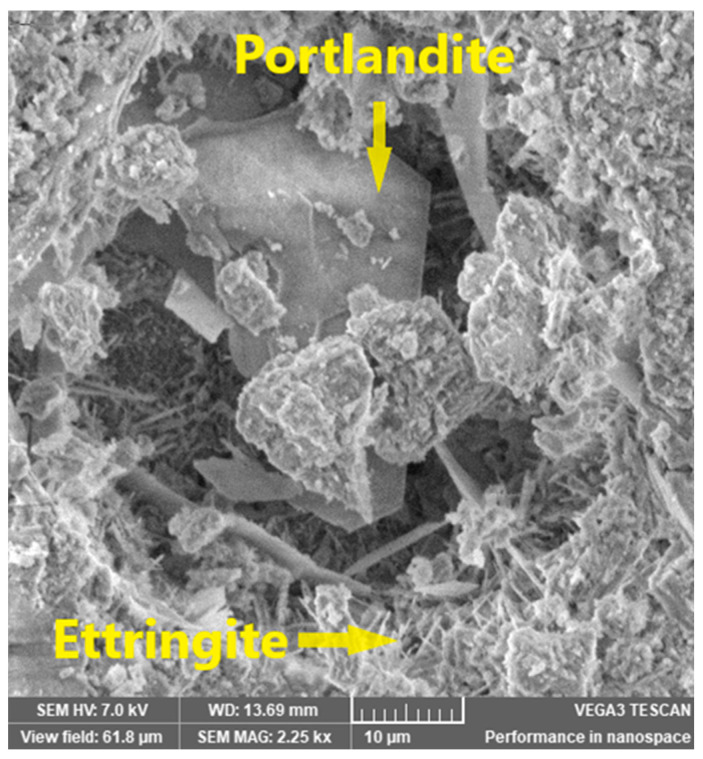
Identification of ettringite and portlandite in CP sample.

The surface of the CP sample was scanned at high magnitude (2.25 k×) (Figure 5) in order to validate the presence of ettringite and portlandite detected in the XRD analysis.

### 3.4. Nuclear Magnetic Resonance Spectroscopy (NMR)

**Figure 6:**
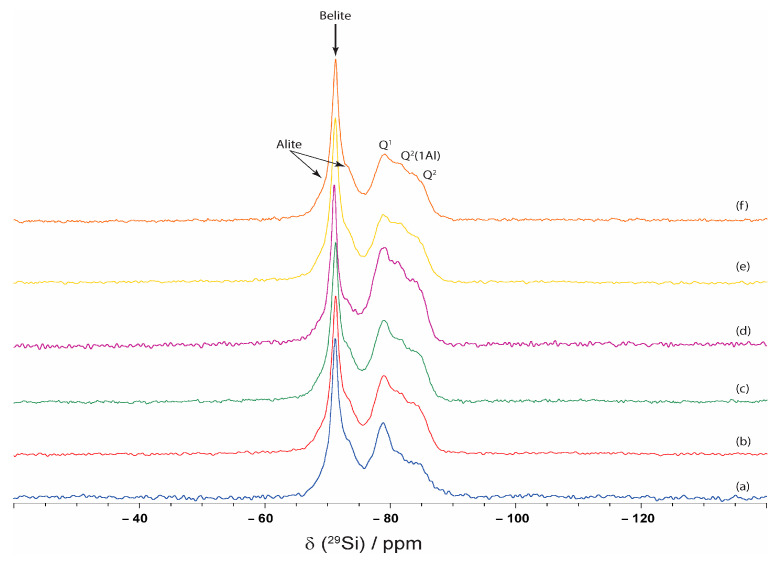
^29^Si NMR spectra of (a) CP, (b) CPSR3, (c) CPSR41, (d) CPW, (e) CPSR3W, and (f) CPSR41W samples.

**Figure 7:**
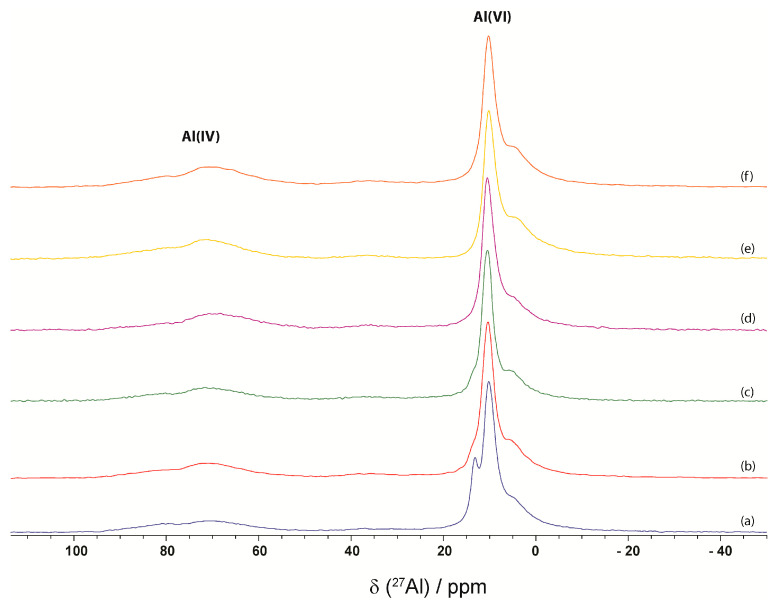
^27^Al NMR spectra of (a) CP, (b) CPSR3, (c) CPSR41, (d) CPW, (e) CPSR3W, and (f) CPSR41W samples.

The NMR spectra of CP, CPSR3, CPSR41, CP1W, CPSR3W, and CPSR41W samples are presented in Figure 6 (^29^Si NMR spectra) and Figure 7 (^27^Al NMR spectra).

The ^29^Si MAS NMR spectra for all samples show o broad spectral line at −71.2 ppm (between −66 ppm and −76 ppm) attributed to the belite and alite primary phase [55]. This spectral line corresponds to the unhydrated clinker. The differences in the unhydrated material (spectra around −71.2 ppm for ^29^Si) in all samples are given by the different amounts of C_3_S and C_2_S, which react with water to produce hydration products in the presence of VSW and superplasticizers. The second very broad spectral line between −75 and −90 ppm corresponds to C–S–H. All the samples contain Q^1^, Q^2^ (1Al), and Q^2^ structural elements [55]. In samples with VSW and superplasticizers, the content of Q^2^ (1Al) and Q^2^ structural elements compared with the content of Q^1^ structural elements increased. The presence of VSW has a positive effect on the hydration of the cement; large amounts of hydration products are obtained, which increases the content of Q^2^ (1Al) and Q^2^. The superplasticizers, combined with VSW, further increase this content as superplasticizers disperse the cement grains, which have a positive effect on the obtained hydration products.

^27^Al MAS NMR spectra are presented in Figure 7 with a clear separation between the Al(IV) and Al(VI) regions. The Al(IV) region presents two small and very broad peaks: at 80.5 ppm corresponding to unreacted clinker phases and 70 ppm corresponding to impurities in the C–S–H gel [56] in the CP, CPSR3, CPSR41, and CPWSR3. The amount of oxygen vacancies in NMR is very small; the peak at about 35 ppm, corresponding to the Al(V), is present in all the samples [48]. In the case of CPW and CPWSR41 present in the Al(IV) region, a small and very broad peak at 70 ppm corresponding to impurities in the C–S–H gel is observed [56]. The Al(VI) region is characterized by three spectral lines: at 13 ppm corresponding to the AFt phase, which is present only in spectra of CP, CPSR3, and CPSR41 and absent in spectra of CPW, CPWSR3, and CPWSR41; at 10 ppm corresponding to AFm phase, which is present in all spectra; and at 5 ppm corresponding to third aluminate hydrate (T) phase, which is also present in all spectra [57,58].

### 3.5. Mechanical Strength

**Figure 8:**
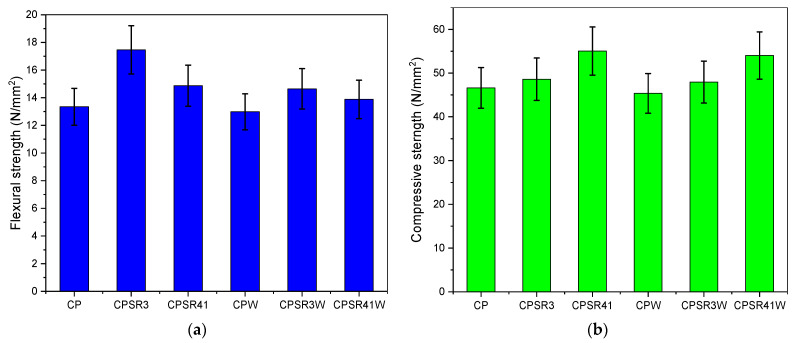
Mechanical properties: (**a**) flexural strength and (**b**) compressive strength of CP, CPSR3, CPSR41, CPW, CPSR3W, and CPSR41W samples.

The mechanical strength (flexural and compressive strength) of the CP, CPSR3, CPSR41, CPW, CPSR3W, and CPSR41W samples is presented in Figure 8a,b. In the presence of VSW (CPW sample), the mechanical properties decrease due to increased water-to-cement ratio, which negatively affects the cement paste properties. The mechanical properties of CPSR3W and CPSR41W containing VSW and superplasticizers increase compared with CP. The highest flexural strength (14.64 N/mm^2^) is obtained for the cement paste with VSW and SR3, while the compressive strength is the highest (54.03 N/mm^2^) in cement paste with VSW and SR41. The superplasticizers disperse the cement grains and vine shoot waste, resulting in a higher quantity of hydrated cement, which produces a higher amount of hydration products, increasing the strength of the CP samples with superplasticizers [31]. Similar results were obtained by Tayeh et al. by using rice husk waste and by dos Santos et al. by using short sugarcane bagasse fibers [17,19].

The obtained results show an improved structure of cement paste with 1% VSW with a lower water-to-cement ratio by adding 1% superplasticizers. The improved structure of the cement paste with 1% VSW and 1% superplasticizers identified by FT-IR, XRD, and SEM is confirmed by the mechanical strength obtained by flexural and compressive determination. FT-IR shows a slight decrease of the –OH band in samples containing plasticizer and plasticizer-waste mixture, suggesting that free lime is converted to carbonates, affecting the mechanical properties. The increase in the strength of the cement paste with VSW is correlated with the higher degree of crystallinity in samples with superplasticizers. Existence of ettringite and portlandite detected in XRD analysis was validated using SEM-EDX, and their presence increase the mechanical properties. The growth of the hydration products observed using ^29^Si and ^27^Al MAS NMR can also be a factor for the increase in the mechanical strength of the samples.

## 4. Conclusions

This study aimed to obtain an improved material 1% VSW with a lower water-to-cement ratio by adding 1% superplasticizers. In the sample with VSW, FT-IR spectra revealed cement paste with similar intensity of the peaks ∼960 cm^−1^, which confirm that the addition of VSW does not delay the formation of calcium hydroxide and of C–S–H, and a slight decrease of the –OH band in samples containing superplasticizers was seen, which suggests that free lime is converted to carbonates. XRD showed a slightly lower intensity of C_2_S and C_3_S diffraction peaks in the presence of VSW due to the higher consumption, and there were no significant differences in the crystalline phases in addition to superplasticizers. The degree of crystallinity (DC) varies between 71.0 and 78.1% for the investigated samples, with the highest values being remarked for the samples with superplasticizers, e.g., CPSR3 (78.1%) and CPSR41 (76.2%). The homogeneity, defects, and variations in the chemical composition of the examined samples were investigated using SEM-EDX. The values obtained for the studied samples’ C/S ratio that are greater than 2.1 that showed the presence of other Ca-based compounds like calcite and portlandite, which support the findings from XRD patterns. ^29^Si MAS NMR spectra show that the content of Q^2^ (1Al) and Q^2^ structural elements compared with the content of Q^1^ structural element increase in samples with VSW and superplasticizers. The presence of VSW has a positive effect on the hydration of the cement; large amounts of hydration products are obtained, while superplasticizers in combination with VSW further increase the hydration products because superplasticizers disperse the cement grains, which have a positive effect on the obtained hydration products. The Al(IV) region presents two small and very broad peaks: at 70 ppm, corresponding to impurities in the C–S–H gel, and at 80.5 ppm, corresponding to unreacted clinker phases. The Al(VI) region is characterized by three spectral lines: at 13 ppm, corresponding to AFt phase, which is present only in spectra of CP, CPSR3, and CPSR41 and absent in spectra of CPW, CPWSR3, and CPWSR41; at about 10 ppm, the peak corresponding to AFm phase, which is present in all spectra; and at 5 ppm, the peak corresponding to third aluminate hydrate (T) phase, which is also present in all spectra. Higher mechanical strength was obtained in the case of using VSW and superplasticizers than in the case of using only VSW. A more dense and compact structure was obtained using VSW with a smaller water/cement ratio in cement paste. A lower water/cement ratio in cement paste with VSW can be obtained by using superplasticizers. The VSW combined with superplasticizers can be used in cement-based materials in wide applications.

## Figures and Tables

**Table 1:** Sample preparation.

Sample Code	Raw Materials	Water-to- Cement Ratio	Superplasticizer -to-Cement Ratio	Vine Shoot Waste -to-Cement Ratio
CP	CP	0.30	–	–
CPSR3	CP + SR3	0.30	0.01	–
CPSR41	CP + SR41	0.30	0.01	–
CPW	CP + VSW	0.40	–	0.01
CPSR3W	CP + VSW + SR3	0.30	0.01	0.01
CPSR41W	CP + VSW + SR41	0.30	0.01	0.01

**Table 2:** Phase fraction (%) and the degree of crystallinity (%) of CP, CPSR3, CPSR41, CPW, CPSR3W, and CPSR41W samples.

Samples	CP	CPSR3	CPSR41	CPW	CPSR3W	CPSR41W
C_2_S	+++	+++	+++	+++	+++	+++
C_3_S	+++	+++	+++	++	+++	+++
Portlandite (C–H)	++	++	++	+++	++	++
Calcium silicate hydrate (C–S–H)	++	++	++	++	++	++
Calcite	+		+	+	+	+
Ettringite	+		+	+	+	+
DC	74.5	78.1	76.2	71.0	75.5	73.7

+++ major phases (>20%), ++ minor phases (5–10%), + phases in traces (<5%).

**Table 3:** The measured values for air holes diameter (d) and distance between them (Q) for investigated samples.

Sample	Range of Values for Air Holes	
	d (μm)	Q (μm)
CP	25.07–25.80	369.04
CPSR3	22.64–54.11	478.46–684.89
CPSR41	285.68–512.53	900.25
CPW	17.75–47.27	106.62–667.44
CPSR3W	15.87–46.52	100.22–511.56
CPSR41W	15.65–29.90	103.94–483.42

**Table 4:** Elemental analysis of CP, CPSR3, CPSR41, CPW, CPSR3W, and CPSR41W samples and C/S ratio.

Element	Mass (%)					
	CP	CPSR3	CPSR41	CPW	CPSR3W	CPSR41W
**O**	47.94	50.58	50.42	47.94	49.98	47.35
**Ca**	41.03	39.45	39.73	40.45	40.28	42.14
**Si**	9.10	8.20	8.22	9.38	8.13	8.79
**Al**	1.93	1.76	1.62	1.78	1.62	1.72
**C/S**	**4.58**	**4.81**	**4.83**	**4.31**	**4.95**	**4.79**

## Data Availability

Not applicable.
